# Multi-model inference using mixed effects from a linear regression based genetic algorithm

**DOI:** 10.1186/1471-2105-15-88

**Published:** 2014-03-27

**Authors:** Koen Van der Borght, Geert Verbeke, Herman van Vlijmen

**Affiliations:** 1 Janssen Infectious Diseases-Diagnostics BVBA, B-2340 Beerse, Belgium; 2 Interuniversity Institute for Biostatistics and statistical Bioinformatics, Katholieke Universiteit Leuven, B-3000 Leuven, Belgium; 3 Interuniversity Institute for Biostatistics and statistical Bioinformatics, Universiteit Hasselt, B-3590 Diepenbeek, Belgium

**Keywords:** Variable selection, Linear regression, Genetic algorithm, Mixed-effects model, Multi-model inference

## Abstract

**Background:**

Different high-dimensional regression methodologies exist for the selection of variables to predict a continuous variable. To improve the variable selection in case clustered observations are present in the training data, an extension towards mixed-effects modeling (MM) is requested, but may not always be straightforward to implement.

In this article, we developed such a MM extension (GA-MM-MMI) for the automated variable selection by a linear regression based genetic algorithm (GA) using multi-model inference (MMI). We exemplify our approach by training a linear regression model for prediction of resistance to the integrase inhibitor Raltegravir (RAL) on a genotype-phenotype database, with many integrase mutations as candidate covariates. The genotype-phenotype pairs in this database were derived from a limited number of subjects, with presence of multiple data points from the same subject, and with an intra-class correlation of 0.92.

**Results:**

In generation of the RAL model, we took computational efficiency into account by optimizing the GA parameters one by one, and by using *tournament* selection. To derive the main GA parameters we used 3 times 5-fold cross-validation. The number of integrase mutations to be used as covariates in the mixed effects models was 25 (*chrom.size*). A GA *solution* was found when R^2^_MM_ > 0.95 (*goal.fitness*). We tested three different MMI approaches to combine the results of 100 GA *solutions* into one GA-MM-MMI model. When evaluating the GA-MM-MMI performance on two unseen data sets, a more parsimonious and interpretable model was found (GA-MM-MMI TOP18: mixed-effects model containing the 18 most prevalent mutations in the GA *solutions*, refitted on the training data) with better predictive accuracy (R^2^) in comparison to GA-ordinary least squares (GA-OLS) and Least Absolute Shrinkage and Selection Operator (LASSO).

**Conclusions:**

We have demonstrated improved performance when using GA-MM-MMI for selection of mutations on a genotype-phenotype data set. As we largely automated setting the GA parameters, the method should be applicable on similar datasets with clustered observations.

## Background

In recent studies, classical regression methods for prediction of a continuous variable from a large number of covariates have been extended for the training of a model when the data set is hierarchical in nature [1-4]. In this article we extend our genetic algorithm (GA) variable selection methodology in [5] to allow for clustering in the data. We compare the performance of multi-model inference (MMI) using restricted maximum likelihood (REML) mixed-effects modeling [6,7] (MM) with ordinary least squares regression [8] (OLS) and compare GA-MMI with the commonly used penalized regression method Least Absolute Shrinkage and Selection Operator [9] (LASSO). We also show how to optimally set the GA parameters.

As an example, the training of a linear regression model for prediction of Raltegravir (RAL) resistance (“phenotype”) from mutations in the HIV integrase region (“genotype”) is worked out. The data sets used for training and testing were described in more detail in [5]. The training set consisted of *n =* 991 clonal genotype-phenotype measurements, from multiple clones derived from 153 clinical isolates (on average 5 à 6 clones per isolate) and repeated measurements (on average 3) from 28 site-directed mutants (in-vitro lab created clones with a designed mutational pattern), and the number of candidate mutations for selection was *p =* 322. Two test sets were used: the first consisted of population data of 171 clinical isolates (test set 1), the second consisted of 67 integrase site-directed mutants containing most of the known RAL resistance associated mutational patterns [10] (test set 2). As it was found in [5] that a second order model did not significantly outperform a first order model, we did not consider interaction terms.

The paper is organized as follows. We begin by recalling the Simple Genetic Algorithm for variable selection in OLS linear regression. Then, we introduce GA-MM as an extension for clustered data. Finally, we introduce MMI for estimation of the model parameters, combining the results from multiple GA-MM (or GA-OLS) runs, followed by a short section on how we applied the LASSO method for comparison. In the remainder of the paper, we illustrate our methodology on an example for the predictive modeling of RAL resistance. For this example, we describe in detail how we optimized the GA parameter settings, and we report the results of comparing GA-MM-MMI with LASSO and GA-OLS-MMI. When nominating one ‘best’ model, from all models evaluated in the comparison, we chose the GA-MM-MMI TOP18 model as a sparse model with high biological relevance (17 out of 18 integrase mutations in this model have been confirmed to be associated with resistance [5]), and having better predictive accuracy than LASSO and GA-OLS-MMI models with equal number of mutations selected. Throughout the text of this article GA related terminology is written in italic.

## Methods

### GA-OLS

The Simple Genetic Algorithm, due to John Holland [11-15], is used to evaluate a set P of regression models M with *p*_*sel*_ variables. In GA terminology: P is a *population*, and a model M ∈ P, is called an *individual*, the *p*_*sel*_ model variables determine the *individuals*’ *chromosome*. The number of models in a *population*, |P|, is fixed, as well as the number of variables *p*_*sel*_ in a model M. In GA terminology: |P| is called the *population size (pop.size)*, and *p*_*sel*_ is called the *chromosome size (chrom.size)*. Thus, each regression model M represents a candidate subset of *p*_*sel*_ variables (in GA terminology variables are called *genes*), and a GA *fitness function* has to be defined to identify the better or ‘more *fit*’ *individuals.* In GA-OLS, we used the linear model R^2^ (OLS) goodness-of-fit statistic as *fitness function*. The better the model M fits to the data, the higher R^2^ (with 0 ≤ R^2^ ≤ 1). Models with R^2^ > *goal fitness* are termed *solutions* to the optimization problem. A Darwinian evolution is applied to modify the *population* over several *generations*. The GA finds a *solution* using the search procedure as given in Table 1.

**Table 1:** Simple genetic algorithm

**Step**	**Description**
1	Initialize a random *population* of *pop.size individuals*, goto step 4.
2	Select the more *fit individuals* to form a new *population*.
3	Modify genetic material of the *individuals* in this new *population* by applying genetic operators: *mutation* and *cross-over*.
4	Evaluate *fitness* of the population. If no *solution* found goto step 2, else end.

In step 3 of Table 1, the *mutation* genetic operator alters a *gene* (replacing it with another *gene* from the pool of candidate *genes*) in a *chromosome* with probability *Pm*. The *crossover* genetic operator re-combines the genotypes of two *individuals*. The probability of an *individual* to be selected for *crossover* is *Pc*. The key in the optimization is to keep a good balance between selective pressure (Table 1 step 2) and genetic diversity (Table 1 step 3). The GA run is completed when an *individual* is found with *fitness* > *goal fitness.* When no *solution* is found within a maximum number of *generations* (*max.generations*), the GA run is halted. For step 2 of Table 1, we used *tournament* selection as detailed in Section **II** (Results and discussion). Also, *elitism* is used, meaning that the best *chromosome* (highest R^2^) is passed through to the next *generation*, with a probability *Pe*.

The running of the GA is done multiple times to generate a set S of *solutions*. A ranking by importance can then be made for all variables based on their frequency in S.

### GA-MM

Although OLS parameter estimates are known to be unbiased when neglecting the correlation structure [6], in this article we want to evaluate whether using a mixed model for the GA models, using a random subject effect in addition to the fixed effects (variables as in the OLS model), can improve the interpretability or performance of the final linear regression model, derived with MMI (next section).

The GA-MM methodology makes use of the Simple Genetic Algorithm (Table 1), completely analogous to GA-OLS, producing a ranking of variables by their frequency in a set S of GA *solutions*. However, there is no single commonly used definition for the R^2^ statistic as is the case for OLS [16,17]. Several definitions have been suggested that all have different interpretations in the presence of correlated errors. Here, we used the marginal R^2^_MM_ definition from [18], quantifying the variance explained by the fixed effects. As new data will originate from other subjects than those used for the training of the model, the random effects cannot be used for prediction. In [1] it has also been described that conditional R^2^ (variance explained by the entire model, including the random effects) should not be used for fixed-effect variable selection. For us, the main motivation for using R^2^_MM_ was that the MM can be fitted using REML, resulting in better estimates for the variance components, needed in the estimation of the fixed effects, especially in models with many fixed effects [7].

In the example, for predicting the RAL phenotype *y* from the integrase clonal genotype *x* ∈ [0, 1]^*p*^, the mixed model *M* uses one random effect/ cluster factor *α*_*i*_ (clones are clustered per clinical isolate/ site-directed mutant):

$$y_{\mathrm{ij}}=\beta_{0}+\sum_{k=1}^{p}\beta_{k}x_{\mathrm{kij}}+\alpha_{i}+\epsilon_{\mathrm{ij}},$$

with *β*_0_ the intercept, and *y*_*ij*_ the j-th response of cluster i,

$$\alpha_{i}\sim N\left(0,\sigma_{\alpha }^{2}\right),$$

and

$$\epsilon_{\mathrm{ij}}\sim N\left(0,\sigma_{\epsilon }^{2}\right)$$

If *x*_*k*_ ∉ *M*: *β*_*k*_ ≡ 0.

The marginal R^2^ is calculated as:

$$R_{\mathrm{MM}}^{2}=\frac{\sigma_{f}^{2}}{\sigma_{f}^{2}+\sigma_{\alpha }^{2}+\sigma_{\epsilon }^{2}},$$

where $\sigma_{f}^{2}$ is the variance calculated from the fixed effects *β*_*k*_:

$$\sigma_{f}^{2}=\mathrm{var}\left(\sum_{k=1}^{p}\beta_{k}x_{\mathrm{kij}}\right),$$

$$\sigma_{\alpha }^{2}$$

is the between-cluster variance, and $\sigma_{\epsilon }^{2}$ is the within-cluster variance.

The intra-class correlation: $\mathrm{ICC}=\frac{\sigma_{\alpha }^{2}}{\sigma_{\alpha }^{2}+\sigma_{\epsilon }^{2}}$ for the model without fixed effects was 0.92, showing very strong within-cluster correlation, and suggesting that accounting for this correlation may improve the performance of our model.

### GA-MMI

In [19,20] it has been described that, when the number of samples in the training data is small, making inference from a single best model, e.g., produced with stepwise regression, leads to the inclusion of noise variables. Here, we used MMI to combine the information from the GA *solutions* into a final model for making predictions. As a GA run is stopped as soon as the *goal fitness* (calculated in section **VI** (Results and discussion)) is achieved (Table 1, step 4), GA *solutions* were ‘equally *fit*’. Thus, we used equal weighting of the GA *solutions* in the MMI. In [6] it was shown that for stepwise regression using an information criterion for selection – as we used in [5] for deriving a consensus model from the GA ranking of variable frequencies – one should for MM use the biased ML estimators. An advantage of using MMI in combination with GA-MM is that REML can still be used. Thus, using MMI, we could make a fair comparison between GA-OLS and GA-MM.

For estimation of the parameters for the final model, we used the following three MMI approaches on the GA *solutions*:

1. Refitting for a TOP selection of the GA ranking: from the GA-ranking, the variables with highest frequencies were retained for the final model, which was then refitted using OLS/MM.

2. Averaging of parameter estimates ${\hat{\beta }}_{k}$ using all GA *solutions* (${\hat{\beta }}_{k}\equiv 0$, if *x*_*k*_ not in GA *solution*) (MMI1):

β¯=k∑s=1Sβ^ksS,

with |S| the number of GA *solutions.*

3. Averaging of parameter estimates ${\hat{\beta }}_{k}$ using GA *solutions* where ${\hat{\beta }}_{k}\neq 0$ (MMI2):

β¯=k∑s=1Sβ^ksM∈S|xk∈M.

For the model averaging in 2 and 3, *parameters*${\hat{\beta }}_{k}$ were (re-)fitted using OLS/MM for all *m* variables with presence at least once in a GA *solution* or for a TOP selection of variables in the GA ranking only.

### LASSO

LASSO [9] is a regularization method that performs variable selection by constraining the size of the coefficients, also called shrinkage. By applying an L1 absolute value penalty, regression coefficients are ‘shrunk’ towards zero, forcing some of the regression coefficients to zero. Using the R package glmselect 1.9-3 [21], for the described example in this paper we performed variable selection using the LASSO technique on the clonal genotype-phenotype database returning a LASSO ranking of variables (solution path) as selected by decreasing the amount of penalty applied. Besides using the shrinkage coefficients for variable estimation (default LASSO) we also applied OLS and MM to the LASSO selected variables (post-LASSO [22]).

## Results and discussion

### GA parameter settings

We optimized the GA parameters one by one in the order (**I** - > **VI**) as described below, and taking computational efficiency into account (see Additional file 1). *Tournament selection* was used as selection method to form a new *population* of more *fit individuals*. GA parameters *Pm* and *Pc* were optimized together using a meta-GA. *Pe* and *pop.size* were fixed in advance and were not optimized. *Pe* was set to conserve the best *chromosome* in three consecutive *generations*, followed by a *generation* where the probability of keeping the best *chromosome* was set to 20%. *Pop.size* was set equal to 20. To set the main GA parameters: *max.generations, chrom.size, and num.runs* we used cross-validation (Additional file 1 point 7).

For running the GA, we used the R package GALGO 1.0.11 [23]. After inspection of the R^2^_CV_ results, with exception of *goal.fitness*, we took the same optimized GA parameters values for GA-OLS and GA-MM (for the model comparison): *pop.size =* 20, *chrom.size =* 25, *Pm =* 0.1, *Pc =* 0.6, *Pe =* (1,1,1,0.2), *max.generations =* 500, *tournament.size = 10, num.solutions = 100, goal.fitness.ols = 0.957, and goal.fitness.mm = 0.95.* In Additional file 2 is the R code we used to derive these settings and to run GA-MM-MMI.

#### **I.** Meta-GA for selection of *Pm* and *Pc*

**Figure 1:**
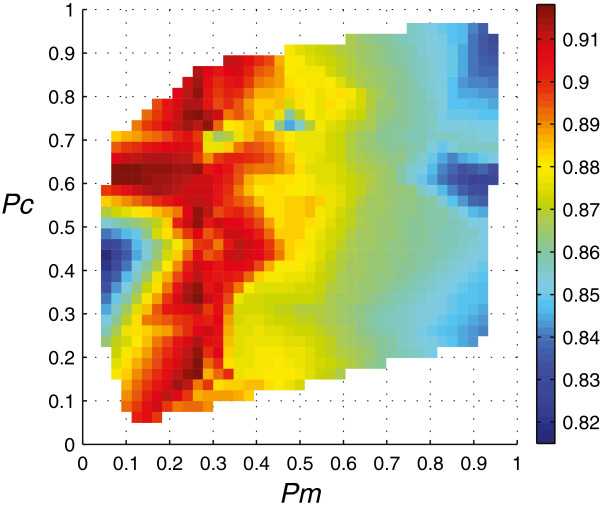
**GA parameter: *mutation *probability (*Pm*) and *cross-over *probability (*Pc*).** R^2^*fitness* landscape from meta-GA.

For the meta-GA optimization of the parameters *Pm* and *Pc* (Table 2), we used the R package gaoptim 1.0 with the default settings (except for *pop.size*_*meta*_*= 20*, instead of 100 (default)) [24]. GA-OLS was used as the meta-GA *fitness function* returning the R^2^ from the best *chromosome* for the (*Pm*, *Pc*) combinations. Different random numbers were generated for each of the GA-OLS runs, thus the same real-valued combination (*Pm*, *Pc*) with multiple presence in the meta-GA population did not give the same *fitness* result. The *fitness* landscape from 2000 (*pop.size*_*meta*_ × *num.generations*_*meta*_) points is shown in Figure 1.

**Table 2:** **Meta-GA optimization of *Pm *** **and *Pc***

***GA***	***CHOSEN***	***PRE-SET***	***BEING OPTIMIZED***
***GA-OLS***	*pop.size = 20*	*chrom.size = 15*	*Pm ∈ [0,1]*
	*Pe = (1,1,1,0.2)*	*max.generations = 100*	*Pc ∈ [0,1]*
		*num.runs = 1*	
		*goal.fitness = 1*	
		*tournament.size = 10*	
***metaGA***	*pop.size*_*meta*_*= 20*		
	*num.generations*_*meta*_*= 100*		
	*Pm*_*meta*_*= 0.01*		
	*Pc*_*meta*_*= 0.9*		
	*Pe*_*meta*_*= 0.4*		

*Crossover* was a fairly weak genetic operator as can be seen from the red band in Figure 1. Oppositely, the *mutation* genetic operator was a strong operator and was best taken in the range [0.1, 0.4]. The meta-GA converged at (*Pm*,*Pc*) = (0.258,0.372). For further evaluation in Section **II**, we also selected (0.1,0.6) and (0.2,0.6) located in the largest dark red area in Figure 1 (R^2^ > 0.91).

#### **II.***Tournament* selection

*Tournament* selection [15,25] is a selection method to bias the selection towards the more *fit individuals. Pop.size tournaments* are organized with k randomly selected *chromosomes*. The winner of a *tournament* is the *chromosome* with the best *fitness* (highest R^2^). The *pop.size tournament* winners become the new *population*. Selection pressure, the degree to which better *individuals* are favoured, is increased when the *tournament* size is increased, as the winner from a large *tournament* will, on average, have a higher *fitness* than the winner of a small *tournament*.

**Figure 2:**
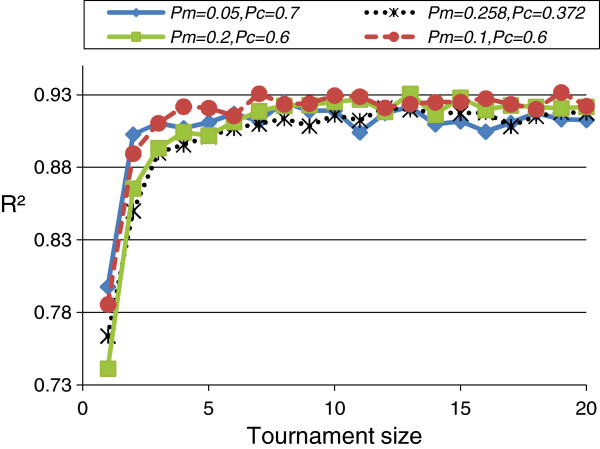
**GA parameter: *tournament *size.** Mean R^2^(OLS) from best *chromosomes* (from 10 runs) on the training data for *tournament* selection with *tournament.size* 1–20 (*pop.size*).

In the optimization (Table 3), all *tournament* sizes 1 ≤ k ≤ *pop.size* were considered. From section **I**, we selected the following (*Pm*,*Pc*) combinations for evaluation: (0.1,0.6), (0.2,0.6), and (0.258,0.372). We also considered (0.05,0.7), the (*Pm*,*Pc*) combination used in [5]. From Figure 2, to improve the R^2^(OLS) performance the *tournament.size k* should be taken > 2. We chose to continue to use *k = 10* (as pre-set in section **I**). Slightly better R^2^ performance was seen for the (*Pm*,*Pc*) combinations (0.1,0.6) and (0.2,0.6). The former was chosen for reasons of computational efficiency.

**Table 3:** **GA parameter settings to evaluate *tournament.size *** **and ( *Pm *, *Pc *)**

***GA***	***CHOSEN***	***PRE-SET***	***BEING OPTIMIZED***
***GA-OLS***	*pop.size = 20*	*chrom.size = 15*	*tournament.size ∈ {1,…,20}*
	*Pe = (1,1,1,0.2)*	*max.generations = 100*	*(Pm,Pc) ∈{(0.1,0.6);(0.2,0.6);*
		*num.runs = 10*	*(0.258,0.372);*
		*goal.fitness = 1*	*(0.05,0.7)}*

#### **III.** Maximum number of *generations*

In Table 4, the GA settings for evaluating *max.generations* are summarized. Evaluation was done for both GA-OLS and GA-MM, calculating $R_{\mathrm{CV}}^{2}$ as the mean from 3 repetitions of 5-fold cross-validation: $R_{\mathrm{CV}}^{2}=\sum_{i=1}^{3}R_{\mathrm{CV},i}^{2}/3$where $R_{\mathrm{CV},i}^{2}$ was calculated as the correlation between the phenotype measurements of all observations in the database (contained exactly once in *test set T*_*ij*_*, j = 1…5*) and their mean prediction (MMI1) of the 10 best *chromosomes* from GA-OLS/GA-MM (trained on *train set TR*_*ij*_ containing 4/5 of the subjects).

**Table 4:** **GA parameter settings to evaluate *max.generations***

***GA***	***CHOSEN***	***PRE-SET***	***BEING OPTIMIZED***
***GA-OLS***	*pop.size = 20*	*chrom.size = 15*	*max.generations ∈ {100,200,300,400,500}*
***GA-MM***	*Pe = (1,1,1,0.2)*	*num.runs = 10*	
	*Pm = 0.1*	*goal.fitness = 1*	
	*Pc = 0.6*		
	*tournament.size = 10*		

**Figure 3:**
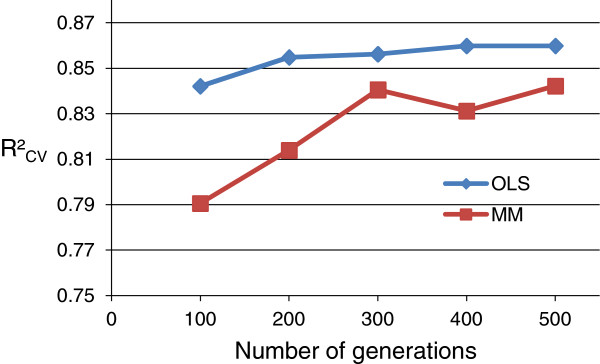
**GA parameter: number of *generations*.** R^2^_CV_ from mean prediction of best *chromosomes* from 10 runs (3 × 5-fold CV) (MMI1).

From Figure 3, it can be seen that, while the improvement in R^2^_CV_ when increasing *max.generations* from 100 to 300 was larger for GA-MM than for GA-OLS, the R^2^_CV_ performance for GA-MM was found to be lower than for GA-OLS. Stabilization of R^2^_CV_ was seen for both GA-OLS and GA-MM for *num.generations* ≥ 400. We chose *max.generations =* 400 to be used further in the sections **IV** and **V**. Note that for the pre-set *goal fitness =* 1, *max.generations* was the number of *generations* always executed. For the model comparison, with the *goal fitness* calculated (Section **VI**), we set *max.generations =* 500 for both GA-OLS and GA-MM.

#### **IV.***Chromosome* size

In Table 5 the GA settings for evaluating *chrom.size* are presented*.* Analogously as for *num.generations*, evaluation was done for GA-OLS as well as GA-MM, using 3 × 5-fold cross-validation (see section **III**).

**Table 5:** **GA parameter settings to evaluate *chrom.size***

***GA***	***CHOSEN***	***PRE-SET***	***BEING OPTIMIZED***
***GA-OLS***	*pop.size = 20*	*num.runs = 10*	*chrom.size ∈ {5,10,15,20,25,30}*
***GA-MM***	*Pe = (1,1,1,0.2)*	*goal.fitness = 1*	
	*Pm = 0.1*		
	*Pc = 0.6*		
	*tournament.size = 10*		
	*max.generations = 400*		

**Figure 4:**
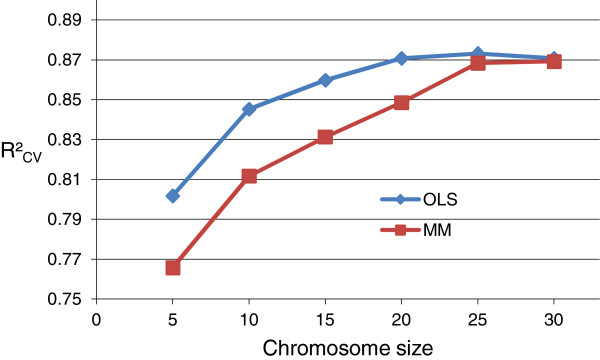
**GA parameter: *chromosome *size.** R^2^_CV_ from mean prediction of best *chromosomes* from 10 runs (3 × 5-fold CV) (MMI1).

The R^2^_CV_ performance is shown in Figure 4. Stabilization in performance was seen for both GA-OLS and GA-MM for *chrom.size* ≥ 25. We chose *chrom.size =* 25 to be used further. Thus, after optimizing *chrom.size*, the GA-MM performance was now equal to the GA-OLS performance (R^2^_CV_ = 0.87).

#### **V.** Number of GA runs

The GA settings for evaluating *num.runs* are shown in Table 6*.* Analogously as for *max.generations* and *chrom.size*, evaluation was done for both GA-OLS and GA-MM using 3 × 5-fold cross-validation (see section **III**).

**Table 6:** **GA parameter settings to evaluate *num.runs***

***GA***	***CHOSEN***	***PRE-SET***	***BEING OPTIMIZED***
***GA-OLS***	*pop.size = 20*	*goal.fitness = 1*	*num.runs ∈ {1,10,20,50,100,500}*
***GA-MM***	*chrom.size = 25*		
	*Pe = (1,1,1,0.2)*		
	*Pm = 0.1*		
	*Pc = 0.6*		
	*tournament.size = 10*		
	*max.generations = 400*		

**Figure 5:**
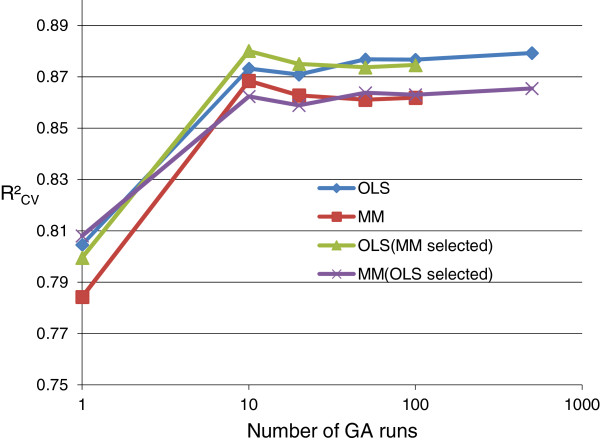
**GA parameter: number of GA runs.** R^2^_CV_ from mean prediction of best *chromosomes* from *num.runs* (3 × 5-fold CV) (MMI1).

In Figure 5 the R^2^_CV_ performance is shown using all best *chromosomes* from *num.runs* in the model averaging (MMI1) (cf. sections **III** and **IV**), including the cases where the GA variable selection is done with MM and re-estimation of the variables in the MMI is done with OLS and vice versa. A similar R^2^ performance was observed when using GA-OLS or GA-MM for the variable selection. However, a higher R^2^_CV_ performance was observed when using OLS for estimation of the best *chromosome* parameters. The R^2^_CV_ performance was stable for *num.runs* ≥ 10. When increasing *num.runs* from 100 to 500 for GA-OLS variable selection*,* only a slight increase in R^2^_CV_ performance was seen.

**Figure 6:**
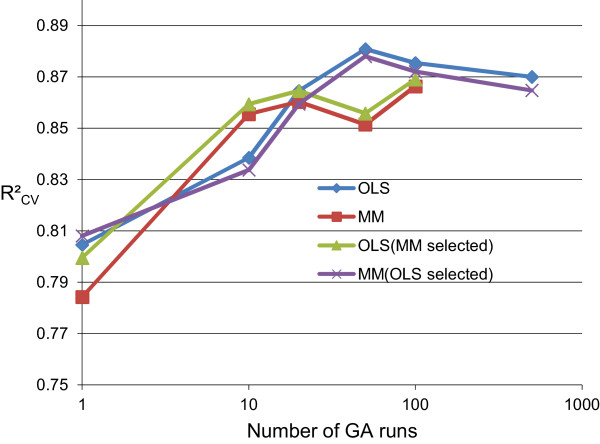
**GA parameter: number of GA runs.** R^2^_CV_ from prediction of ‘best’ best *chromosome* from *num.runs* (3 × 5-fold CV).

In Figure 6 the R^2^_CV_ performance is shown using only the ‘best’ best *chromosome* from *num.runs* for prediction in the cross-validation. Overall, a similar R^2^_CV_ performance was observed when using OLS or MM for estimation of the ‘best’ best *chromosome* parameters, and using GA-MM or GA-OLS for variable selection.

**Figure 7:**
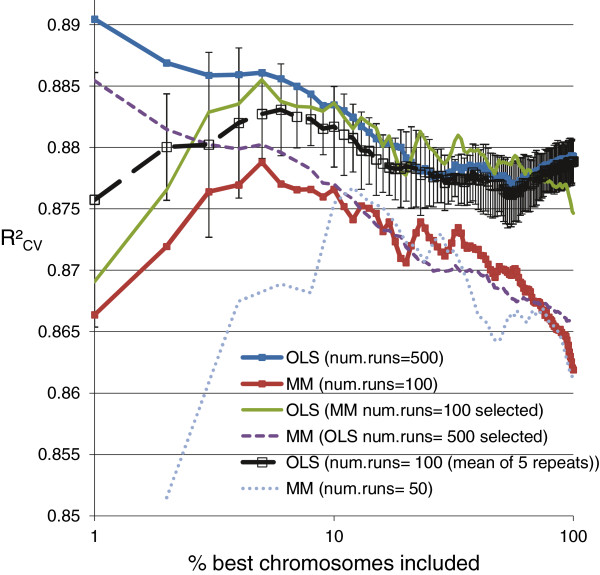
**GA parameter: number of GA runs.** R^2^_CV_ from mean prediction of ‘x% best’ best *chromosomes* from *num.runs* (3 × 5-fold CV) (MMI1).

In Figure 7, the ‘x% best’ best *chromosomes* (shown on the x-axis in log scale) were used in the model averaging (MMI1). Evaluation was done for *num.runs =* 100 or 500 and *num.runs =* 50 or 100 for GA-OLS and GA-MM, respectively. For GA-OLS, the highest R^2^_CV_ was 0.89 and was obtained when including the ‘five best’ best *chromosomes* (top 1% *chromosomes* with highest R^2^(OLS) from *num.runs =* 500). Also, for GA-MM, including the ‘five best’ best *chromosomes* (top 5% of *num.runs =* 100 with highest R^2^_MM_) gave the highest R^2^: 0.879 and 0.885 for MMI-MM and MMI-OLS, respectively. Thus, both for GA-OLS-MMI and GA-MM-MMI inclusion of the ‘five best’ best *chromosomes* yielded an improvement in R^2^_CV_ performance in comparison to using all best chromosomes (Figure 5) or ‘the best’ best *chromosome* (Figure 6). As previously noted from Figure 5, also a better R^2^_CV_ performance was again found using OLS estimation of parameters than when using MM estimation. For GA-OLS *num.runs = 100* was repeated 5 times (splitting the best *chromosomes* as available from *num.runs =* 500 in five consecutive parts for evaluation using MMI1). The mean curve of these 5 repeats is shown, together with the 95% confidence interval error bars. The peak of this mean curve is at ‘6% best’ best *chromosomes* included. The GA-MM curve with MMI-OLS estimation (*num.runs =* 100) is situated within these error bars. Thus, for *num.runs =* 100 GA-MM and GA-OLS perform equally well in selecting variables for the model in the cross-validation. For GA-OLS the R^2^_CV_ performance using the ‘five best’ best *chromosomes* using *num.runs =* 500 is better than when using *num.runs =* 100. Therefore, calculation of the *goal fitness* for GA-OLS in section **VI** was done from the *num.runs =* 500 best *chromosomes*. For calculation of the *goal fitness* for GA-MM, *num.runs =* 100 was used. Note that, once the goal fitness was calculated, *num.runs* was set to NA (not applicable). Instead, the model comparison will be based on *num.solutions =* 100 (number of best *chromosomes* with R^2^ > *goal.fitness*) for both GA-OLS and GA-MM.

#### **VI.***Goal fitness*

As derived from Figure 7, for calculating the *goal fitness* we considered the *fitness* of the ‘5 best’ best *chromosomes*: this is the top 1% (of *num.runs =* 500) for GA-OLS and the top 5% (of *num.runs =* 100) for GA-MM. For each of the 15 (3 × 5) CV training sets we calculated the non-parametric one-sided (1-*p*,1-*α*) tolerance upper limit [26] on the R^2^*fitness* distribution of best *chromosomes* from *num.runs* with *p =* 1% and *p =* 5% for GA-OLS and GA-MM, respectively, and *α =* 0.05 (95% confidence). The interpretation is that with confidence level 1-*α* not more than (100 × *p*)% of the best *chromosomes* have R^2^*fitness* values exceeding this limit. To be able to calculate these tolerance limits the requested number of runs was ${\left[\frac{\ln\left(\alpha \right)}{\ln\left(1-p\right)}\right]}_{+}$[26]. This requirement was met for *num.runs = 100* ≥ 59, and *num.runs = 500* ≥ 299 for GA-MM and GA-OLS, respectively. The goal fitness was then calculated as the mean of the CV tolerance upper limits:

$$\mathrm{goal}.\mathrm{fitness}=\sum_{i=1}^{15}\mathrm{tol}.\mathrm{uppe}r_{i}/15,$$

which equals *goal.fitness =* 0.957 for GA-OLS and *goal.fitness =* 0.95 for GA-MM. For the calculation we used the R package tolerance 0.5.2 [27].

### GA-OLS *vs.* GA-MM: variable selection

GA-OLS and GA-MM variable selection were performed on the clonal genotype-phenotype database using the GA parameters as specified in the above sections. The percentage of runs that failed reaching the *goal.fitness* with *max.generations =* 500 was 16% and 23.1% for GA-OLS and GA-MM, respectively.

**Figure 8:**
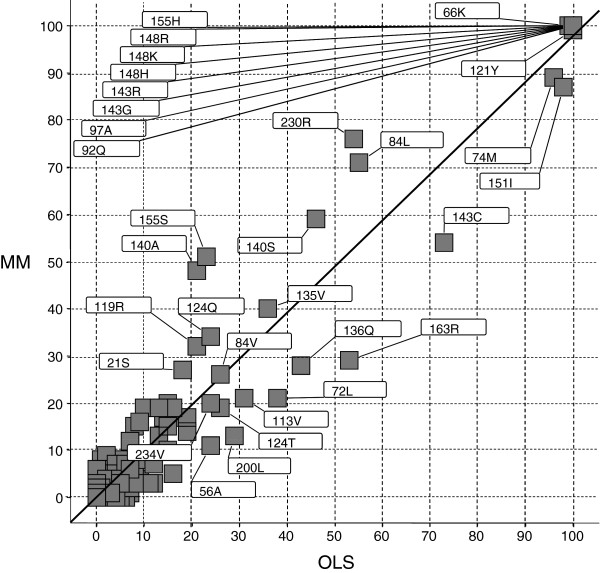
**Pearson correlation of GA frequency rankings using MM *vs. *OLS (R**^**2 **^**= 0.95).** Mutations with frequency > 20 for OLS or MM are labeled.

Figure 8 shows the relation between the frequency of the variables selected in the GA using OLS and MM. While frequency differences were clearly observed (e.g. for 74M, 151I, 230R, 84L, 140S, 143C, 155S, and 140A), a strong correlation was obtained (R^2^ = 0.95). Eight integrase mutations were selected as variables in all 100 GA *solutions* for both GA-OLS and GA-MM: 92Q, 97A, 143G, 143R, 148H, 148K, 148R, 155H. This number would possibly be lower when increasing *num.solutions,* leading to non-selection for a few GA *solutions*. This was now already the case with *num.runs =* 100 for 66K (always selected by GA-MM, 99/100 selected by GA-OLS) and 121Y (always selected by GA-OLS, 99/100 selected by GA-MM).

### GA-OLS and GA-MM variable selection *vs.* LASSO

**Figure 9:**
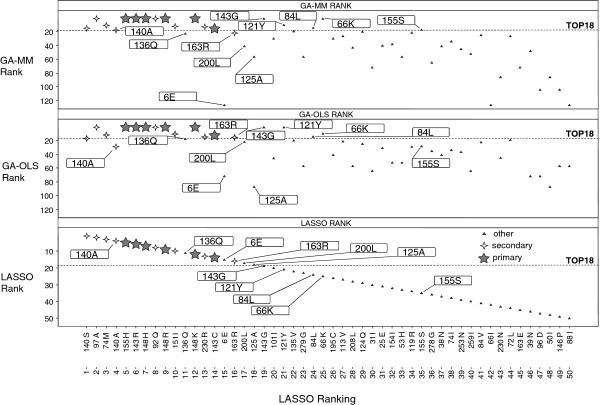
**LASSO ranking (50 mutations) compared with GA-MM and GA-OLS.** A reference line is shown for the top 18 ranked mutations. Using the RAL product label [10], mutations are indicated as primary/secondary or other.

Figure 9 shows the comparison of the GA-OLS and GA-MM ranking with the LASSO top 50 ranking of variables, shown on the x-axis. The variables selected are integrase mutations, indicated as primary/secondary/other. Primary and secondary mutations have been associated with RAL resistance in [10]. Note that whereas a single primary mutation causes RAL resistance, the effect on resistance of secondary mutations not in a combination with a primary mutation is minor [5]. In Figure 9, most of the primary and secondary mutations had a high ranking for GA-OLS, GA-MM and LASSO. However, some of the ‘other’ mutations such as 66K, 121Y, 143G and 155S with presence in one or more of the publically available genotypic algorithms: ANRS (http://www.hivfrenchresistance.org), Rega (http://regaweb.med.kuleuven.be), and Stanford (http://hivdb.stanford.edu), had a lower ranking for LASSO. We note that 66K, 121Y and 155S were introduced in the database as site-directed-mutants not in a combination with other mutations [5], and LASSO was less sensitive in selecting these. Another observation was that for LASSO, the secondary mutations 140A and 140S were ranked higher than the primary mutations. Also, mutations not listed by any of the public algorithms such as 6E, 125A, and 200L, had a higher ranking for LASSO in comparison to GA-OLS and GA-MM. Oppositely, one of the “other” integrase mutations ranked higher by GA-OLS and GA-MM, and not listed by any of the public genotypic algorithms was 84L. In [5] we already discussed that its selection may result from a more complex interaction of three secondary mutations with which 84L co-occurred in the clones of one clinical isolate.

When we compared the GA-OLS ranking with the GA-MM ranking (Figures 8 and 9), a relatively low ranking was seen e.g., for GA-OLS for 140A and 155S, which favours GA-MM for its interpretation.

### GA-OLS-MMI *vs.* GA-MM-MMI *vs.* LASSO: R^2^ performance on test set 1 and test set 2

In Tables 7 and 8 are the results of the R^2^ performance comparison of GA-OLS-MMI, GA-MM-MMI, and LASSO on the two test sets with *n =* 171 clinical isolates and *n =* 67 site-directed mutants, respectively. Models containing the TOP15-18-21-24-27-30 or ALL variables as selected by LASSO, GA-OLS and GA-MM were considered. Note that as randomness is incorporated in the GA optimization techniques there are more mutations with presence in at least one of the GA *solutions, m =* 193 and *m =* 200 for GA-OLS and GA-MM respectively, compared to *m =* 51 mutations with absolute value of the regression coefficients above zero in the LASSO solution path.

**Table 7:** **R**^**2 **^**performance on test set 1**

***Variable selection***	**LASSO**			**GA-OLS**						**GA-MM**					
***Variable estimation***	**Coef (shrinkage)**	**OLS**	**MM**	**OLS**	**MM**	**MMI1**		**MMI2**		**OLS**	**MM**	**MMI1**		**MMI2**	
						**OLS**	**MM**	**OLS**	**MM**			**OLS**	**MM**	**OLS**	**MM**
**TOP15 variables**	0.815	0.816	0.827	0.830	0.833	0.832	0.838	0.827	0.831	0.834	0.835	0.834	**0.839**	0.829	0.832
**TOP18 variables**	0.816	0.818	0.827	0.830	0.831	0.833	0.838	0.825	0.829	0.832	0.835	0.835	**0.839**	0.829	0.832
**TOP21 variables**	0.819	0.825	0.835	0.821	0.825	0.836	**0.839**	0.819	0.824	0.824	0.826	0.834	0.838	0.819	0.824
**TOP24 variables**	0.820	0.822	0.824	0.819	0.824	0.837	**0.840**	0.818	0.824	0.820	0.821	0.834	0.837	0.817	0.821
**TOP27 variables**	0.827	0.817	0.818	0.827	0.829	0.839	**0.841**	0.822	0.827	0.814	0.820	0.835	0.838	0.814	0.819
**TOP30 variables**	0.828	0.812	0.817	0.821	0.822	0.838	**0.840**	0.819	0.823	na^c^	na^c^	na^c^	na^c^	na^c^	na^c^
**ALL *m *** **variables**^**a**^	0.826	0.795	0.811	na^b^	na^b^	0.840	**0.841**	0.701	0.725	na^b^	na^b^	0.838	0.839	0.713	0.725
	(*m =* 51)					(*m =* 193)						(*m =* 200)			

^a^*m* is the number of variables with presence in the GA *solutions* or with abs(coef) > 0 in the LASSO solution path. ^b^not calculated due to singularity.

^c^no model with exactly 30 mutations.

In bold the highest R^2^ per row is indicated.

**Table 8:** **R**^**2 **^**performance on test set 2**

***Variable selection***	**LASSO**			**GA-OLS**						**GA-MM**					
***Variable estimation***	**Coef (shrinkage)**	**OLS**	**MM**	**OLS**	**MM**	**MMI1**		**MMI2**		**OLS**	**MM**	**MMI1**		**MMI2**	
						**OLS**	**MM**	**OLS**	**MM**			**OLS**	**MM**	**OLS**	**MM**
**TOP15 variables**	0.667	**0.734**	0.712	0.707	0.707	0.708	0.708	0.709	0.710	0.709	0.702	0.705	0.696	0.706	0.698
**TOP18 variables**	0.690	0.731	0.713	0.721	0.718	0.716	0.714	0.722	0.719	0.768	**0.770**	0.742	0.742	0.747	0.750
**TOP21 variables**	0.742	0.760	0.765	0.736	0.730	0.722	0.717	0.732	0.726	**0.777**	0.775	0.746	0.744	0.751	0.752
**TOP24 variables**	0.745	**0.771**	0.768	0.732	0.728	0.720	0.716	0.727	0.723	0.762	0.761	0.743	0.740	0.748	0.749
**TOP27 variables**	0.767	**0.788**	**0.788**	0.721	0.725	0.720	0.717	0.732	0.726	0.770	0.768	0.744	0.741	0.758	0.755
**TOP30 variables**	0.777	**0.789**	0.787	0.768	0.772	0.731	0.729	0.747	0.743	na^c^	na^c^	na^c^	na^c^	na^c^	na^c^
**ALL *m *** **variables**^**a**^	**0.787**	0.770	0.776	na^b^	na^b^	0.733	0.729	0.741	0.733	na^b^	na^b^	0.747	0.745	0.754	0.749
	(*m =* 51)					(*m =* 193)						(*m =* 200)			

^a^*m* is the number of variables with presence in the GA *solutions* or with abs(coef) > 0 in the LASSO solution path. ^b^not calculated due to singularity.

^c^no model with exactly 30 mutations.

In bold the highest R^2^ per row is indicated.

On test set 1, using MM for the variable estimation had a slightly better R^2^ performance than using OLS, for all models considered. Note that this was not the case in the cross-validation (section **V**) where OLS R^2^_CV_ performance was higher, possibly due to the inclusion of multiple clinical isolates from the same patient. However, as patient information was not given for the training set, we could not take this into account. For the TOP15/TOP18 models containing the smallest number of variables, the best performance was seen for GA-MM-MMI1 (R^2^ = 0.839). For the TOP21- > ALL models with more variables considered, the best performance was seen for GA-OLS-MMI1 (R^2^ = 0.839-0.841). When estimating ALL GA-OLS/GA-MM variables, the worst performance was seen for MMI2 (R^2^ = 0.701-0.725) where noise variables were clearly overweighted. For LASSO, the best R^2^ performance on test set 1 was obtained using MM for the variable estimation for the TOP15- > TOP24 selection of variables (R^2^ = 0.824-0.835). For LASSO TOP27- > ALL, the best R^2^ performance was obtained using the LASSO shrinkage coefficients (R^2^ = 0.826-0.828).

On test set 2, for the sparse models the best performance was observed for LASSO-OLS TOP15 (R^2^ = 0.734), GA-MM-MM TOP18 (R^2^ = 0.770), and GA-MM-OLS TOP21 (R^2^ = 0.777). For the TOP21- > ALL models, the best performance was seen for LASSO (R^2^ = 0.771-0.789). In contrast to the results for test set 1, the MMI2 R^2^ performance was now found to be higher than for MMI1, for the GA-OLS/MM models. The reason is that while test set 1 consisted of clinical samples, with 82.5% not containing any of the primary RAL resistance mutations [5], test set 2 consisted of site-directed mutants containing most of the known resistance patterns but lacking any noise variables as found in clinical samples. Nevertheless, on test set 2, the GA-MM R^2^ values were found to be better than for GA-OLS, confirming that a better selection of variables as made by GA-MM (cf. the above two sections) led to a better performance on unseen data.

Therefore, on the example training set in this article we would favour the GA-MM-MMI TOP18 model. Based on the performance on test set 2, for the MMI variable estimation re-fitting using MM may be preferred over MMI1-MM.

## Conclusions

In this article, we extended our GA variable selection methodology to mixed models which account for clustering in the data. Using cross-validation, we optimized the GA parameter settings taking also computational efficiency into account. For the worked-out example, all settings could be taken equal for GA-OLS and GA-MM, with exception of *goal.fitness* for which we used a marginal R^2^ definition. The model parameters for prediction could then be estimated using MMI-MM (REML) on the GA *solutions* obtained from 100 GA runs. When testing LASSO, GA-OLS and GA-MM on two unseen data sets, all methods had good performance. When imposing a parsimony restriction for better interpretability of the model, the GA-MM-MMI TOP18 model had better predictive accuracy (R^2^) than GA-OLS and LASSO.

In summary, we belief that GA-MM-MMI is a direct approach to derive a sparse and interpretable model for making predictions with good accuracy on small data sets with clustered observations and a large number of candidate variables, where chance of overfitting with standard regression techniques is high.

### Availability and requirements

Project name: GA-MM-MMI.

Project home page: http://sourceforge.net/projects/ga-mm-mmi.

Operating system: Platform independent.

Programming language: R ≥ 2.15.2, perl, MATLAB.

Other requirements: requires galgo 1.0.11 [23].

License: GNU GPL.

Any restrictions to use by non-academics: none.

## Competing interests

The authors declare that they have no competing interests

## Authors’ contributions

KVdB developed the GA-MM-MMI approach, performed all analyses and wrote the manuscript. GV conceived of the study, assisted in its design, and revised the manuscript. HvV assisted in study design. All authors read and approved the final manuscript.

## Supplementary Material

Additional file 1 **GA parameter settings and computational efficiency.** Computational efficiency is taken into account by setting the GA parameters one by one: **I** and **II**: *Pm* and *Pc*, **II***tournament.size*, **III***max.generations*, **IV***chrom.size*, **V***num.runs* (needed until *goal.fitness* is set in **VI**). Pre-set values are used for the parameters to be optimized later in the process. For deriving the GA parameters in **III** - > **V** we used 3 × 5-fold cross-validation. Click here for file

Additional file 2 **R code.** GA-MM-MMI source code in R programming language (R ≥ 2.15.2 from http://cran.r-project.org). Click here for file
