# Supplementary material for: Multi-model inference using mixed effects from a linear regression based genetic algorithm
Source: BMC Bioinformatics. 2014 Mar 27;15:88. doi: 10.1186/1471-2105-15-88 (PMC3987104; doi:10.1186/1471-2105-15-88)
Supplement: Additional file 1 — GA parameter settings and computational efficiency. Computational efficiency is taken into account by setting the GA parameters one by one: I and II: Pm and Pc, IItournament.size, IIImax.generations, IVchrom.size, Vnum.runs (needed until goal.fitness is set in VI). Pre-set values are used for the parameters to be optimized later in the process. For deriving the GA parameters in III - > V we used 3 × 5-fold cross-validation. [file 1471-2105-15-88-S1.pdf]

GA parameter settings and computational efficiency.

1. *Tournament* selection was used (explained in section **II**). In section **I**, *tournament.size*=10 was pre-set.
2. Determining *Pm* and *Pc (and tournament.size)* was done on the training data, using GA-OLS only. (Note that for the other GA parameters 3 x 5-fold cross-validation was used for both GA-OLS and GA-MM, see below).
3. The following GA parameters were fixed in advance and were not optimized: *pop.size*=20, and *Pe*=(1,1,1,0.2). The best *chromosome* was conserved in three consecutive *generations*, followed by a *generation* where the probability of keeping the best *chromosome* was set to 20%. Generally, passing the best *chromosome* (having the highest R^2) through to the next *generation*, makes the GA efficient, as it means that the R^2 can only improve in the next *generations*. However, sometimes lowering *Pe* allows for a faster escape from a poor local peak (best *chromosome*, having a low R^2).
4. In the sections **I**-**II** we took *max.generations*= 100.
5. In the sections **I**-**III** *chrom.size* was set equal to 15 (which is equal to the number of resistance mutations listed in the RAL drug label [10] selected for the first order linear regression model in [5]).
6. In section **I** and in the sections **II**-**IV**, we took *num.runs*= 1 and *num.runs*= 10, respectively (where the GA is run for the maximum number of *generations* (*goal.fitness*=1)).
7. 3 x 5-fold cross-validation was used in determining *max.generations*, *chrom.size*, and *num.runs.* The observations in the clonal genotype-phenotype database were divided into 5 groups, such that all observations with the same clinical isolate/ site-directed mutant ID were found in the same group. Five times, a GA ranking of variables was generated on 4/5 groups, each time leaving out one group for testing. The cross-validated performance (R^2_{CV}) was then calculated as the R^2 correlation between predictions from genotypes in the test data and the corresponding phenotype measurements for these genotypes in the database.

The 5-fold cross-validation was repeated 3 times, with a different division into 5 sets. Thus, a total of 15 training sets (and 15 test sets) were created on which GA-OLS and GA-MM were trained (in the sections **III**-**V**). For computational efficiency, R^2_{CV} performance was evaluated for a limited set of GA parameter training values only: 100-200-300-400-500 (*max.generations*), 5-10-15-20-25-30 (*chrom.size*), and 20-50-100-500 (*num.runs*). Because computation of MM was approximately 4 times less time efficient than calculation of OLS, *num.runs*=500 was evaluated for GA-OLS only.
